# Dietary Supplementation With Chinese Herbal Residues or Their Fermented Products Modifies the Colonic Microbiota, Bacterial Metabolites, and Expression of Genes Related to Colon Barrier Function in Weaned Piglets

**DOI:** 10.3389/fmicb.2018.03181

**Published:** 2018-12-21

**Authors:** Jiayi Su, Qian Zhu, Yue Zhao, Li Han, Yulong Yin, Francois Blachier, Zhanbin Wang, Xiangfeng Kong

**Affiliations:** ^1^Hunan Provincial Key Laboratory of Animal Nutritional Physiology and Metabolic Process, Key Laboratory of Agro-ecological Processes in Subtropical Region, National Engineering Laboratory for Pollution Control and Waste Utilization in Livestock and Poultry Production, Institute of Subtropical Agriculture, Chinese Academy of Sciences, Changsha, China; ^2^College of Animal Science and Technology, Henan University of Science and Technology, Luoyang, China; ^3^Nutrition Physiology and Ingestive Behavior, UMR 914 INRA/AgroParisTech/Universite Paris-Saclay, Paris, France

**Keywords:** bacterial metabolites, Chinese herbal residues, colon barrier function, fermentation, microbiota, weaned piglets

## Abstract

To explore the feasibility of dietary Chinese herbal residue (CHR) supplementation in swine production with the objective of valorization, we examined the effects of dietary supplementation with CHR or fermented CHR products on the colonic ecosystem (i.e., microbiota composition, luminal bacterial metabolites, and expression of genes related to the intestinal barrier function in weaned piglets). We randomly assigned 120 piglets to one of four dietary treatment groups: a blank control group, CHR group (dose of supplement 4 kg/t), fermented CHR group (dose of supplement 4 kg/t), and a positive control group (supplemented with 0.04 kg/t virginiamycin, 0.2 kg/t colistin, and 3000 mg/kg zinc 0.04 kg/t virginiamycin, 0.2 kg/t colistin, and 3000 mg/kg zinc oxide). Our results indicate that dietary supplementation with CHR increased (*P* < 0.05) the mRNA level corresponding to *E*-cadherin compared with that observed in the other three groups, increased (*P* < 0.05) the mRNA level corresponding to zonula occludens-1, and decreased (*P* < 0.05) the quantity of *Bifidobacterium* spp. When compared with the blank control group. Dietary supplementation with fermented CHR decreased (*P* < 0.05) the concentration of indole when compared to the positive control group; increased (*P* < 0.05) the concentrations of short-chain fatty acids compared with the values measured in the CHR group, as well as the mRNA levels corresponding to interleukin 1 alpha, interleukin 2, and tumor necrosis factor alpha. However, supplementation with fermented CHR decreased (*P* < 0.05) interleukin 12 levels when compared with the blank control group. Collectively, these findings suggest that dietary supplementation with CHR or fermented CHR modifies the gut environment of weaned piglets.

## Introduction

Weaning is a critical stage for piglets, as this process is associated with alterations in the architecture and function of the gut and changes in the enteric microbiota (Boudry et al., 2004). Weaned piglets often exhibit underdeveloped immune systems, digestive disorders, and post-weaning diarrhea (Liu et al., 2016), all these events decreasing growth performance and causing economic loss for the swine industry (Amezcua et al., 2002). Antibiotics and the pharmacological addition of ZnO in the diets have long been used to solve post-weaning problems (Barton, 2000). However, their continuous use and misuse have led to the emergence of drug resistant bacteria, the risk of residual antibiotics in animal products, and the release of Zn-residues in the environment (Schwarz et al., 2001). Therefore, many alternatives to antibiotics and ZnO have been explored in response to this situation (Hu et al., 2013; Kong et al., 2014).

Among potential alternatives, Chinese herbs (CH) are one of the most promising candidates (Kong et al., 2011). Recent findings indicated that the use of CH as a dietary additive is able to enhance gastrointestinal health indicators in weaned piglets (Kong et al., 2007b; Ding et al., 2011). Moreover, dietary supplementation with Chinese herbal ultra-fine powder enhances both cellular and humoral immunity in early weaned piglets (Kong et al., 2007a). Chinese herbal residues (CHR) are produced during CH production and processing. The use of partial CH extraction technology leads to CHR containing high levels of nutrients and bioactive ingredients, and the resulting CHR exhibits the same efficiency as CH with regards to enhanced gastrointestinal health, increased nutrient absorption, and immune system stimulation. However, the presence of cell walls in CHR results in the presence of a large number of active components that may act on the gut ecosystem. Therefore, limited utilization of CHR by animals and environmental pollution from byproducts represent two major drawbacks of using CHR as a feed supplement.

The process adopted for CHR fermentation involves inoculation of only one or a few types of probiotics from beneficial microbiota to produce fermented CHR. These products contain compounds originating from the microbiota, including bioactive ingredients, and bacterial metabolites (Sun et al., 2011). In addition, the levels of anti-nutritional factors and potentially toxic components are reduced, and numerous functional secondary metabolites are produced, thus resulting in better characteristics of fermented CHR compared to non-fermented CHR (Wen et al., 2013).

The production of CHR and fermented CHR remains limited despite the increasing popularity of herbal fermentation. Our previous study showed that fermented CHR can improve growth performance and digestion in weaned piglets up to a certain extent, without, however, any significant impact on the rate of diarrhea and intestinal morphology (Su et al., 2016). Recent studies have highlighted the pivotal role of the gut microbiota in animal health and diseases (Tremaroli and Bäckhed, 2012). We hypothesized that dietary supplementation with CHR or fermented CHR may exert beneficial effects on the colonic ecosystem, and subsequently tested the effects of these compounds on the colonic microbiota, bacterial metabolite concentration, and gene expression related to the intestinal barrier function in weaned piglets. A reduction in pathogen concentration may result in a reduction in toxic bacterial metabolite production, and of competition with host for nutrient utilization, thus improving host weight gain. Therefore, the aim of this study was to further test this hypothesis and to evaluate the interest of CHR and their fermented products utilization as feedstuff or feed additives for swine production.

## Materials and Methods

### Ethics Statement

The experimental design and procedures in this study were reviewed and approved by the Animal Care and Use Committee of the Institute of Subtropical Agriculture, Chinese Academy of Sciences. The processing of animal experiments and sample collection strictly followed the relevant guidelines.

### Preparation and Composition of Fermented CHR

The fermentation substrates used in the present study, including residues of Radix Rehmanniae Preparata, Fructus Crataegi, Pericarpium Citri Reticulatae, Fructus Hordei Germinatus, and Radix Glycyrrhizae, were supplied by Hunan Sunaccord Biological Technical Co., Ltd., China. The ratio of Radix Rehmanniae Preparata residues, Fructus Crataegi residues, Pericarpium Citri Reticulatae residues, Fructus Hordei Germinatus residues, and Radix Glycyrrhizae residues was 4:2:2:1:1.

After sterilization, mixed CHR containing 40–60% water was inoculated with *Bacillus subtilis* and yeast, incubated at 28–32°C for 22–26 h in anaerobic conditions, inoculated with *Lactobacillus* and *Bifidobacterium* and incubated at 31–34°C for 24 h in anaerobic conditions. The ratio of *B. subtilis*, yeast, *Lactobacillus*, and *Bifidobacterium* was 5:2:2:1, and the concentration of live bacteria was > 2 × 10^10^ colony forming units per gram (CFU/g). After fermentation, the fermented products were dried in a vacuum, pulverized, and packed for use. The analyzed contents (%) of nutrients based on dry matter (with the exception of dry matter) in CHR and fermented CHR are listed in Table 1.

**Table 1:** Nutrient levels of CHR and fermented CHR.

Nutrient component	Content/%	
	CHR	Fermented CHR
DM	93.4	95.49
GE	13.15	16.11
CP	7.75	11.51
CF	2.64	2.22
EE	6.39	4.93
Ash	25.66	10.97
ADF	49.51	26.64
NDF	40.50	33.11

### Animals, Housing and Treatment

This study used a total of 120 piglets (Duroc × Landrace × Large White), weaned at 21 days of age with an average body weight of 6.12 ± 0.05 kg. Piglets were randomly assigned to one of four treatment groups, with five replicates per group and six piglets per replicate. The four treatment groups consisted of a blank control group (basal diet), CHR group (basal diet supplemented with 4 kg/t CHR), fermented CHR group (basal diet supplemented with 4 kg/t fermented CHR), and a positive control group (the basal diet supplemented with 0.04 kg/t virginiamycin, 0.2 kg/t colistin, and 3000 mg/kg zinc oxide). All pigs were housed in 2.0 m × 2.5 m pens with hard plastic slatted flooring, and pigs had *ad libitum* access to drinking water and experimental diets. Each pen was equipped with a stainless-steel feeder and a nipple drinker. The room temperature was maintained at 25–27°C. The composition and nutrient levels of the basal diet met the nutritional needs that National Research Council (2012) recommended for nursery piglets, which are shown in Table 2. The experiments lasted for 28 days.

**Table 2:** Composition and nutrient levels of basal diet (fed-basis).

Ingredients	Ratio/%	Nutrient component^2^	Content/%
Corn	22.0	DE/ (MJ/kg)	14.23
Broken rice	25.0	CP	18.02
Wheat flour	12.0	EE	4.37
Glucose	3.0	ASH	3.82
Soybean meal (46% CP)	10.5	CF	2.31
Puffed soybean	10.0	Ca	0.80
Fermented soybean meal	2.5	TP	0.55
Fish meal	3.0	AP	0.40
Low-protein whey powder	5.0	Lys	1.38
Egg powder	0.5	Met	0.49
Soybean oil	1.0	Thr	0.87
Citric acid	1.5	Trp	0.24
Premix^1^	4.0		
Total	100.0		

*^*1*^The premix provided the following per kg of the diet: VA 6 200 IU, VD_*3*_ 700 IU, VE 88 IU, VK 4.4 mg, VB_*2*_ 8.8 mg, Pantothenate 24.2 mg, nicotinic acid 33 mg, Chloride choline 330 mg, Cu 10 mg, Zn 100 mg, Fe 145 mg, Mn 40 mg, Se 0.1 mg, I 0.3 mg. ^*2*^Nutrient contents are calculated values.*

### Sample Collection and Preparation

At the end of the 28-day experimental period and 12 h after the last feeding, a medium-sized piglet from each replicate was sacrificed using general anesthesia (Kong et al., 2007b). After colon recovery, the intestinal contents from each colon (10 cm from the posterior to the ileocecal valve) were collected and stored at -20°C for analysis of short-chain fatty acids (SCFAs), indoles, skatoles, ammonia (considered as the sum of NH_3_ and NH_4_^+^), and the composition of microbiota. Colon tissue samples (approximately 2 cm) were collected, washed with cold physiological saline, immediately frozen in liquid nitrogen and stored at -80°C until further analysis by RNA extraction was conducted.

### DNA Extraction and Analysis of the Quantity of Colonic Microbiota

Total microbial DNA was extracted and purified using a QIAamp DNA Stool Kit (Qiagen, Hilden, Germany) and stored at -80°C. The 16S rRNA gene sequences of Bacteroidetes, *Bifidobacterium* spp., *Clostridium* cluster IV, *Clostridium* cluster XIVa, *Escherichia coli*, Firmicutes, *Lactobacillus*, and total bacteria (Raveh-Sadka et al., 2015) were cloned into the pMD19-T vector. Gene sequences were amplified from total colonic DNA using the primers listed in Table 3. A total of eight clones with 16S rRNA gene sequences belonging to different taxa were used as templates to test primer specificity. Standard curves were constructed with DNA from representative species for a concentration range from 10^2^ to 10^10^ DNA copies/mL using a Lightcycler 480II instrument (Applied Biosystems). General microbial DNA extracted from colonic contents and specific DNA from recombinant microbiota were quantified using RT-PCR. The reaction conditions were as follows: 2 min at 50°C; an initial denaturation step at 95°C for 5 min; 40 cycles of denaturation at 94°C for 20 s, primer annealing at a species-specific temperature for 30 s, and primer extension at 60°C for 1 min (Decroos et al., 2006).

**Table 3:** 16S rRNA gene-targeted group-specific primers used in this study.

Items	Sequence (5′-3′)	Amplicon length (bp)
Bacteroidetes	F: GGARCATGTGGTTTAATTCGATGAT	126
	R: AGCTGACGACAACCATGCAG	
Bifidobacterium spp.	F: TCGCGTCYGGTGTGAAAG	128
	R: GGTGTTCTTCCCGATATCTACA	
Clostridium cluster IV	F: GCACAAGCAGTGGAGT	240
	R: CTTCCTCCGTTTTGTCAA	
Clostridium cluster XIVa	F: CGGTACCTGACTAAGAAGC	189
	R: AGTTTYATTCTTGCGAACG	
Escherichia coli	F: CATGCCGCGTGTATGAAGAA	95
	R: CGGGTAACGTCAATGAGCAAA	
Firmicutes	F: GGAGYATGTGGTTTAATTCGAAGCA	126
	R: AGCTGACGACAACCATGCAC	
Lactobacillus	F: AGCAGTAGGGAATCTTCCA	345
	R: ATTCCACCGCTACACATG	
Total bacteria	F: GTGSTGCAYGGYYGTCGTCA	123
	R: ACGTCRTCCMCNCCTTCCTC	

### Analysis of the Colonic Concentrations of Bacterial Metabolites

The contents of each colon were recovered by expulsion, homogenized, and centrifuged at 1000 × *g* for 10 min (Zhou et al., 2012). A mixture of supernatant fluid and 25% metaphosphoric acid solution (1:0.25 mL) was prepared to determine SCFAs by gas chromatography (Zhou et al., 2014). The concentration of ammonia in the supernatant fluid was measured at a wavelength of 550 nm using a UV-2450 spectrophotometer (Shimadzu, Japan) (Kong et al., 2014). Indoles and skatoles were analyzed as described previously (Kong et al., 2016).

### Analysis of the Levels of mRNAs Corresponding to Epithelial Cell Proteins, Cytokines, and TLR4 Signaling Pathway Proteins in Colonic Tissues

Total RNA was isolated from colonic tissues using TRIzol reagent (Invitrogen, Carlsbad, CA, United States), and samples were treated with DNAse. The RNA quality was checked using 1% agarose gel electrophoresis followed by staining with 10 μg/mL ethidium bromide. The OD260/OD280 ratio of extracted RNA was between 1.8 and 2.0. Reverse transcription was performed using a Prime Script RT Reagent Kit with gDNA Eraser (Takara, Dalian, China). The mRNA levels of the selected genes in the colon were determined by real-time quantitative (RT-qPCR) as described previously (Li et al., 2015; Wan et al., 2017, Wan et al., 2018b). Selected genes were corresponding to the following proteins: epithelial cell proteins, including *E*-cadherin, occludin, and zonula occludens-1 (ZO-1); anti-inflammatory cytokines, including interleukin-4 (IL-4), IL-10 and transforming growth factor-beta-1 (TGF-β1); proinflammatory cytokines, including granulocyte-macrophage colony-stimulating factor (GM-CSF), IL-1α, IL-1β, IL-2, IL-12, and tumor necrosis factor alpha (TNF-α); toll-like receptor 4 (TLR4) signaling pathway proteins, including cluster of differentiation 14 (CD14) and TLR4. The primers used to amplify these genes are shown in Table 4. Relative expression was reported as a ratio of the expression of the target gene to that of beta-actin (β-actin), and data were expressed relative to the data recorded in basal diet-treated piglets. The relative expression ratio (R) of mRNA was calculated as *R* = 2^-ΔΔCt^ (sample – control), where -ΔΔCt (sample – control) = (Ct_target gene_ – Ct_β-actin_) sample – (Ct_target gene_ – Ct_β-actin_) control. RT-qPCR was performed using a SYBR Green detection kit (Thermo Fisher Scientific, Waltham, MA, United States), and the conditions were as follows: 30 s denaturation at 94°C, 30 s annealing at 60°C, and 30 s extension at 72°C for 40 cycles, followed by a melting curve program (60–99°C with a heating rate of 0.1°C/s and fluorescence measurement). A melting temperature (T_m_) peak of 85 ± 0.8°C was used to determine the specificity of amplification. T_m_ values are reported as the mean of three replicates (Liu et al., 2016).

**Table 4:** Primers used for quantitative reverse transcription PCR.

Items	Sequence (5′-3′)	Length (bp)
β-actin	F: CTGCGGCATCCACGAAACT	147
	R: AGGGCCGTGATCTCCTTCTG	
CD14	F:CCTCAGACTCCGTAATGTG	180
	R: CCGGGATTGTCAGATAGG	
*E*-cadherin	F: GAAGGAGGTGGAGAAGAGGAC	216
	R: AGAGTCATAAGGTGGGGCAGT	
GM-CSF	F: CGGCTGTGATGAATGAAACC	164
	R: GTGCTGCTCATAGTGCTTGG	
IL-1α	F: ACCCGACTGTTTGTGAGTGC	111
	R: TTCCCAGAAGAAGAGGAGACTG	
IL-1β	F: GCTAACTACGGTGACAACAA	196
	R: TCTTCATCGGCTTCTCCACT	
IL-2	F: TGCACTAACCCTTGCACTCA	100
	R: CAACTGTAAATCCAGCAGCAA	
IL-4	F: CCCGAGTGTCAAGTGGCTTA	122
	R: TGATGATGCCGAAATAGCAG	
IL-10	F: GGGCTATTTGTCCTGACTGC	105
	R: GGGCTCCCTAGTTTCTCTTCC	
IL-12	F: ATCTCGGTTGGTGTTGTTCC	103
	R: GGGTATCTCGTCCTCTGTCC	
Occludin	F: ATGCCTCCTCCCCTTTCG	295
	R: CGCCCGTCGTGTAGTCTGTC	
TGF-β1	F: AAGCGGCAACCAAATCTATG	113
	R: CCCGAGAGAGCAATACAGGT	
TNFα	F: ACAGGCCAGCTCCCTCTTAT	102
	R: CCTCGCCCTCCTGAATAAAT	
TLR4	F: CAGATAAGCGAGGCCGTCATT	113
	R: TTGCAGCCCACAAAAAGCA	
ZO-1	F: TACCCTGCGGCTGGAAGA	154
	R: GGACGGGACCTGCTCATAACT	

*GM-CSF, granulocyte-macrophage colony-stimulating factor; IL, interleukin; TGF-β1, transforming growth factor-beta-1; TLR, toll-like receptor; TNFα, tumor necrosis factor alpha; ZO-1, zonula occludens-1.*

### Statistical Analysis

Data were statistically analyzed by one-way ANOVA using SPSS 17.0 software (SPSS, Inc., Chicago, IL, United States). Data are presented as means ± SEM, and *P*-values < 0.05 indicate statistical significance.

## Results

### Colonic Microbiota in Piglets

The quantities of Bacteroidetes, *Clostridium* cluster IV, *E. coli*, Firmicutes, *Lactobacillus*, and total bacteria in the colon contents did not change significantly (*P* > 0.05) after supplementation with CHR or fermented CHR. The quantity of *Clostridium* cluster XIVa decreased (*P* < 0.05) in the positive control group compared with the other three groups (Table 5). The quantities of *Bifidobacterium* spp. in the positive control and CHR groups were significantly lower (*P* < 0.05) than in the blank control group.

**Table 5:** Effects of dietary supplementation with Chinese herbal residues (CHR) or fermented CHR on colonic microbiota quantities in weaned piglets (*n* = 5; lg copies/g).

Items	Blank control group	CHR group	Fermented CHR group	Positive control group
*Bacteroidetes*	9.97 ± 0.33	9.79 ± 0.11	9.55 ± 0.44	9.98 ± 0.21
*Bifidobacterium* spp.	4.62 ± 0.11^a^	3.68 ± 0.12^b^	4.16 ± 0.50^ab^	3.58 ± 0.14^b^
*Clostridium* cluster *IV*	9.20 ± 0.29	8.66 ± 0.11	7.53 ± 1.24	8.40 ± 0.14
*Clostridium* cluster *XIVα*	8.42 ± 0.29^a^	7.13 ± 0.44^ab^	8.64 ± 0.81^a^	6.59 ± 0.45^b^
*Escherichia coli*	7.42 ± 0.56	6.92 ± 0.32	6.89 ± 1.34	6.20 ± 0.31
*Firmicutes*	9.56 ± 0.23	9.45 ± 0.22	9.39 ± 0.13	9.27 ± 0.16
*Lactobacillus*	7.72 ± 0.36	7.74 ± 0.45	6.01 ± 1.46	6.62 ± 0.14
Total bacteria	10.87 ± 0.18	10.40 ± 0.08	10.32 ± 0.36	10.43 ± 0.16

*Data in the same row with different superscripts differ significantly (*P* < 0.05).*

### Concentrations of Bacterial Metabolites in the Colons of Piglets

As shown in Table 6, there were no significant differences (*P* > 0.05) between colonic luminal concentrations of ammonia and skatoles in the four treatment groups. Dietary supplementation with fermented CHR significantly decreased (*P* < 0.05) the concentration of indoles in the colonic contents compared with that in the positive control group. When compared with the CHR group, the results indicated that fermented CHR increased (*P* < 0.05) the colonic luminal concentrations of straight-chain fatty acids (including acetate, propionate, butyrate, and valerate) and branched-chain fatty acids (including isobutyrate and isovalerate).

**Table 6:** Effects of dietary supplementation with Chinese herbal residues (CHR) or fermented CHR on bacterial metabolite concentrations in the colonic content in weaned piglets (*n* = 5; mg/g).

Items	Blank control group	CHR group	Fermented CHR group	Positive control group
Ammonia	10.87 ± 2.09	11.56 ± 1.56	12.79 ± 2.22	10.39 ± 1.87
Indole (μg/g)	26.94 ± 6.55^ab^	29.24 ± 5.17^ab^	17.97 ± 3.14^b^	43.48 ± 8.68^a^
Skatole (μg/g)	72.15 ± 26.94	71.00 ± 19.96	58.69 ± 11.26	40.51 ± 7.79
Acetate	7.48 ± 2.14	4.68 ± 1.44	6.60 ± 1.24	7.06 ± 1.62
Propionate	2.64 ± 0.53	2.06 ± 0.21	2.37 ± 0.47	2.99 ± 0.70
Butyrate	1.83 ± 0.38	1.12 ± 0.15	1.46 ± 0.31	1.71 ± 0.52
Valerate	0.46 ± 0.10	0.32 ± 0.03	0.42 ± 0.06	0.36 ± 0.05
Isobutyrate	0.44 ± 0.08	0.31 ± 0.03	0.38 ± 0.05	0.34 ± 0.04
Isovalerate	0.99 ± 0.22	0.67 ± 0.12	0.83 ± 0.04	1.19 ± 0.32
Straight-chain fatty acid	12.40 ± 3.10^ab^	8.19 ± 1.73^b^	10.86 ± 2.07^a^	9.62 ± 1.74^a^
Branched-chain fatty acids	1.43 ± 0.30	0.98 ± 0.15	1.26 ± 0.04	1.25 ± 0.22

*Data in the same row with different superscripts differ significantly (*P* < 0.05). Straight-chain fatty acids, including acetate, propionate, butyrate, and valerate; Branched-chain fatty acids, including isobutyrate and isovalerate.*

### Levels of mRNA Corresponding to Epithelial Cell Proteins, Cytokines, and Proteins of the TLR4 Signaling Pathway in the Colons of Piglets

As shown in Table 7, dietary supplementation with CHR increased (*P* < 0.05) the mRNA level of *E*-cadherin compared with that in the other three groups and increased ZO-1 relative to that in the blank control group. Dietary supplementation with CHR or fermented CHR increased (*P* < 0.05) the mRNA levels of IL-1α, IL-2, and TNF-α, while decreasing (*P* < 0.05) the level of IL-12 compared with that in the blank control group. Piglets fed a diet supplemented with CHR exhibited higher (*P* < 0.05) levels of IL-4 and TGF-β1, but a lower (*P* < 0.05) level of GM-CSF was observed compared with those in the fermented CHR group. The level of CD14 mRNA was significantly higher (*P* < 0.05) in the positive control group than in the other three groups.

**Table 7:** Effects of dietary supplementation with Chinese herbal residues (CHR) or fermented CHR on colon mRNA levels corresponding to epithelial cell proteins, cytokines, and TLR4 signaling pathway in weaned piglets (*n* = 5).

Items	Blank control group	CHR group	Fermented CHR group	Positive control group
CD14	6.05 ± 1.35^b^	3.15 ± 1.44^b^	2.67 ± 0.50^b^	10.58 ± 2.34^a^
*E*-cadherin	0.76 ± 0.19^b^	2.20 ± 0.15^a^	0.96 ± 0.18^b^	0.75 ± 0.12^b^
GM-CSF	0.59 ± 0.17^c^	0.88 ± 0.11^bc^	1.44 ± 0.12^a^	1.22 ± 0.22^ab^
IL-1α	5.04 ± 1.28^b^	11.61 ± 2.37^a^	11.78 ± 1.98^a^	8.51 ± 1.74^ab^
IL-1β	4.05 ± 1.14	3.93 ± 1.01	4.34 ± 0.83	6.18 ± 1.21
IL-2	0.39 ± 0.07^b^	1.65 ± 0.54^a^	2.09 ± 0.52^a^	1.18 ± 0.23^ab^
IL-4	1.50 ± 0.46^b^	3.24 ± 0.72^a^	1.29 ± 0.15^b^	3.68 ± 0.34^a^
IL-10	1.57 ± 0.46	2.32 ± 0.81	2.61 ± 0.65	1.56 ± 0.27
IL-12	0.38 ± 0.13^a^	0.16 ± 0.03^b^	0.12 ± 0.03^b^	0.24 ± 0.03^a^
Occludin	1.77 ± 0.30	2.78 ± 0.54	2.85 ± 0.50	2.76 ± 0.60
TGFβ1	2.20 ± 0.49^ab^	3.26 ± 0.53^a^	2.05 ± 0.15^b^	2.66 ± 0.18^ab^
TNFα	0.74 ± 0.10^b^	1.73 ± 0.44^a^	1.71 ± 0.33^a^	1.06 ± 0.13^ab^
TLR4	3.40 ± 0.92	4.89 ± 0.65	3.08 ± 0.37	4.72 ± 0.55
ZO-1	0.17 ± 0.04^b^	0.29 ± 0.02^a^	0.24 ± 0.03^ab^	0.26 ± 0.03^a^

*Data in the same row with different superscripts differ significantly (*P* < 0.05). GM-CSF, granulocyte-macrophage colony-stimulating factor; IL, interleukin; TGF-β1, transforming growth factor-beta-1; TLR, toll-like receptor; TNFα, tumor necrosis factor alpha; ZO-1, zonula occludens-1.*

## Discussion

Gut microbiota are the resident microorganisms in the digestive tracts of animals and humans, which affect nutrient digestion and the bioconversion of food compounds in the host organisms (Seo et al., 2015). Recently, the intestinal microbiota composition and metabolic activities have emerged as important parameters affecting either positively or negatively “intestinal health” (Blachier et al., 2017). Components such as microbiota composition and diversity and bacterial metabolite concentrations can affect the epithelial integrity, barrier function, immunity, and enteroendocrine peptides. It is known that the intestinal microbiota has genomic characteristics that allow it to use “providential” undigested nutrients in feedstuff. In return, it benefits the host metabolism by providing energy through the production of metabolites that can be utilized and absorbed by colonic cells (Cani and Delzenne, 2007). The intestinal microbiota and associated metabolites affect positively or negatively intestinal barrier permeability, depending on their chemical structure and concentrations, as well as activate immune cells and produce pro- or anti-inflammatory molecules in the gut by cell-associated pattern recognition receptors that recognize molecules unique to the microbiota components or its metabolites (Macpherson and Harris, 2004). Indeed, changes in the luminal environment of the intestinal epithelial cells, following notable modifications in dietary intake, can negatively or positively affect the homeostatic process of colonic epithelia renewal and barrier function (Blachier et al., 2017).

Firmicutes and Bacteroidetes are regarded as the main microbiota phyla in the pig gut, representing approximately 90% of all phylogenetic types (Guo et al., 2008). The present study validated this report by showing quantities of Firmicutes and Bacteroidetes that were similar to the total bacterial quantity. Blais et al. (2015) reported that the most abundant members of Firmicutes, including *Clostridium* clusters IV and XIVa, along with *Lactobacillus* and *Bifidobacterium*, appear beneficial for host health. Although microbiota belonging to *Clostridium* clusters IV and XIVa are more abundant than *Lactobacillus* and *Bifidobacterium*, they have received far less attention. Moreover, current knowledge of the mucosa-associated microbial communities in the colon is limited because studies have focused on the characterization of fecal diversity (Decroos et al., 2006). Therefore, the present study determined the quantities of several important colonic microbiota groups based on real-time PCR. Although these data do not reflect the total bacterial community, our data show that the quantities of all tested microorganisms, especially *Clostridium* cluster XIVa and *Bifidobacterium*, were lower in the positive control group than in the blank control group. This is likely due to the addition of antibiotics and ZnO in the positive control group because these compounds affect the balance of intestinal microbiota. In addition, although solid-state anaerobic fermentation was inoculated with *Lactobacillus* and *Bifidobacterium*, there was no difference in the quantities of these two probiotics with regard to the colon luminal contents between the CHR and fermented CHR groups. It is thus tempting to speculate that fermentation methods explain this result, but further studies outside the scope of the present study are necessary to test such a hypothesis.

The gut microbiota benefits the host by breaking down undigestible or not fully digested nutrients, as well as regulating the immune, endocrine and mesenteric nervous systems (Guarner and Malagelada, 2003). It is well known that the SCFAs are major compounds produced by the microbial fermentation of fibers and resistant starch (Laparra and Sanz, 2009), and to a lower extent, by some amino acids. The large intestine is the main site of SCFA production and absorption in pigs. SCFAs, especially butyrate, are the main energy source for colonocytes (Perrin et al., 2001), and they presumably play an important role in colon epithelium renewal (Nicholson et al., 2012). Butyrate produced by *Clostridium* clusters IV and XIVa is not only used by colonocytes for energy production, but it also effects gene expression, in relationship with the control of colonocyte proliferation and differentiation (Carneiro et al., 2008). Importantly, it is worth to keep in mind that SCFA concentrations represent the net result of microbiota production and colon mucosa absorption.

The results of the present study suggest that dietary supplementation with CHR decreased the butyrate concentration, but it did not significantly modify the quantities of *Clostridium* clusters IV and XIVa. It is likely that in our study a large amount of butyrate was absorbed by colon mucosa, resulting in increased mRNA levels of *E*-cadherin and ZO-1 in colonic tissue. In addition, dietary fibers may be fermented by the gut microbiota to produce health-promoting SCFAs that are believed to modify intestinal barrier function and microbiota composition (Chen et al., 2017). Although numerous types of fibers are present in CHR (crude fiber, 2.64%; acid detergent fiber, 49.51%; and neutral detergent fiber, 40.50% [based on dry matter]), the SCFAs concentration in the colonic contents originating from the CHR group was the lowest. A possible mechanism is that non-digestible fibers reach the large intestine and provide a source of energy for colonocytes through the metabolic activity of the microbiota, which produces SCFAs. Colonocytes then absorb and partially metabolize SCFAs (Konstantinov et al., 2003). This interpretation needs further validation.

The gut microbiota can also catabolize nitrogenous compounds to putrefactive catabolites, such as ammonia, sulfide, bioamines, indoles, and phenols (Blachier et al., 2007). The ammonia concentration in the lumen of the large intestine is primarily attributed to microbiota amino acid deamination and urea hydrolysis (Warren and Newton, 1959), microbiota utilization of ammonia, and ammonia absorption through epithelial cells (Eklou-Lawson et al., 2009; Andriamihaja et al., 2010). Ammonia in excess has been considered as a metabolic troublemaker because of its ability to inhibit mitochondrial oxygen consumption in a dose-dependent manner. In addition, a high concentration of ammonia can inhibit SCFA oxidation in colonic epithelial cells (Cremin et al., 2003). In the present study, there were no significant differences in the concentration of ammonia in the colonic contents recovered from the four treatment groups. This suggests that dietary supplementation with CHR or fermented CHR does not lead to increased protein fermentation in the colon, and thus is not associated with a marked increase of protein-derived amino acid deamination and/or urea hydrolysis in the colonic content. Compared with other amino acids, aromatic amino acids are metabolized and fermented slowly by anaerobes, including *Bacteroides, Lactobacillus, Bifidobacterium*, and *Clostridium* (Jensen et al., 1995). Tryptophan yields indoleacetate and indole, with the former subsequently yielding skatole (Smith and Macfarlane, 1997). Among these bacterial metabolites, indole has been shown to be beneficial for colonic epithelial barrier function by increasing epithelial cell tight junction (TJ) resistance (Le et al., 2005). From an environmental perspective, the odor resulting from indole and skatole emissions during intensive periods of swine production has a negative impact on meat quality and a serious nuisance to people living in close proximity (Le et al., 2005). In the present study, the fermented CHR significantly decreased the concentration of indole in the colonic content, and this could be the result of a decreased production of indole and/or enhanced absorption through the colonic epithelium. Nonetheless, this decrease is presumably detrimental to the colonic epithelium since this particular bacterial metabolite, as discussed above, was shown to contribute to the maintenance of colonic barrier function (Shimada et al., 2013). In contrast, the co-metabolite indoxyl sulfate is considered a uremic toxin and can activate the aryl hydrocarbon receptor (AhR)-mediated transcription of several enzymes belonging to the cytochrome P450 family, with associated negative effects. Additionally, the reduction in the amount of fecal indole released into the environment is potentially beneficial. Further studies, including the measurement of related polluting substances in fecal material, are necessary to fully validate the latter proposition (Zhou et al., 2014), although they are outside the scope of the present study.

The surface of the gut is protected by a layer of epithelial cells covered by mucous layers, which is in constant contact with an abundant population of microbes and their metabolites (Hartsock and Nelson, 2008). The intestinal barrier formed by the epithelial cells and junctional complex, consisting of TJs (including occludin, claudin, and ZO-1), adherens junctions (including *E*-Cadherin and catenins), gap junctions, and desmosomes, excludes the vast majority of these microbes and some of their metabolites from access to the subepithelial cells (Ukena et al., 2007). A previous study reported that microbiota can influence the integrity of the intestinal epithelium, mucosal immunity function, and host health (Cerf-Bensussan and Gaboriau-Routhiau, 2010), and this in turn can affect intestinal barrier function and, consequently, intestinal permeability. Moreover, several pathogens have been shown to modulate the epithelial TJs, and this is achieved via the production of proteins that engage signaling mechanisms in epithelial cells, modulate the actin cytoskeleton, or degrade TJ proteins (Su et al., 2011). Hence, modulation of epithelial cell protein function, particularly by increasing the mRNA levels of *E*-Cadherin, occludin, and ZO-1, is a target for novel therapeutics or prophylactic treatments against a range of diseases involving alterations of the epithelial barrier function (Yang et al., 2015). The mechanism for the increased expression of *E*-cadherin and ZO-1 after dietary CHR supplementation is unknown but may be related to increases in the mRNA levels of anti-inflammatory cytokines, including IL-4, IL-10, and TGF-β1, which in turn affects the mRNA levels of *E*-cadherin and ZO-1. In the present study, the ZO-1 mRNA level in the positive control group increased relative to the blank control group, and this is consistent with a previous finding that zinc supplementation reduced intestinal permeability by enhancing occluding and ZO-1 expression in weaning piglets (Zhang and Guo, 2009).

Cytokines are small peptides that play important roles in the regulation of immune and inflammatory responses (Pié et al., 2004). On one hand, proinflammatory cytokines (e.g., IL-1α, IL-2, and TNF-α) are necessary to initiate the inflammatory response during infection, but the overexpression of such cytokines may lead to pathological responses (Clark, 2007; Zelnickova et al., 2008). Therefore, regulating the expression levels of proinflammatory cytokines could potentially reduce intestinal mucosal inflammation (Liu et al., 2008; Wan et al., 2018a). On the other hand, anti-inflammatory cytokines (e.g., TGFβ1 and IL-4) are capable of inhibiting the overexpression of proinflammatory cytokines and other mediators that could lead to hyperactivation of the immune response in weaned piglets (Clark, 2007; Zelnickova et al., 2008). In addition, the intestinal immune system has been reported to be closely linked to the intestinal microbiota, to the TLR4 signaling pathway, and to the intestinal TJ barrier (Wu and Wu, 2012). For example, *Clostridium* clusters IV and XIVa are capable of promoting the induction of colonic regulatory T cells (Atarashi et al., 2011); and TLR4/CD14 signaling pathways are known to mediate an inflammatory response to microbial stimuli (Takeda and Akira, 2005). Furthermore, most proinflammatory cytokines (TNF-a and IL-1β) induce a pathological opening in the intestinal TJ barrier and increase intestinal epithelial permeability (Ai-Sadi et al., 2009). In the present study, the colons of piglets fed a diet supplemented with CHR exhibited higher levels of IL-1α, IL-2, TNF-α, and IL-4, but a lower level of IL-12. Finally, we proposed that the CHR-induced changes in microbiota composition and metabolic activity act directly on the epithelium or indirectly by activating immune cells that, in turn, affect epithelial function. Additional studies are required to define the complex relationships between these different components.

## Conclusion

In the present study, we show that dietary supplementation with CHR or fermented CHR has no major adverse effects on the colon ecosystem in weaned piglets. Supplementation with CHR modified the expression of genes related to TJ epithelial cell proteins, as well as several cytokines in the colon. The fermented CHR supplementation modified the expression of genes encoding several cytokines and decreased indole concentrations in the colonic content, this latter bacterial metabolites being involved in colon epithelial barrier function. However, it should be kept in mind that this latter compound is also associated with deleterious effects, notably as precursor of the uremic toxin indoxyl sulfate. Although our study demonstrates that diets supplemented with CHR or fermented CHR modify the colon ecosystem in terms of microbiota, bacterial metabolite composition, and gene expression, further study is needed to elucidate the overall effects of such dietary supplementation in terms of beneficial and deleterious effects on the colon and overall piglet health. Finally, future studies investigating the mechanisms of action by which CHR and fermented CHR impact the gut ecosystem should determine the possible causal links between the different parameters measured in our study.

## Author Contributions

JS, QZ, YZ, LH, and XK performed the experiments. JS, YY, FB, ZW, and XK wrote the manuscript. JS and XK performed the statistical analysis. JS, QZ, YZ, and LH fed the animals. All authors reviewed the manuscript.

## Conflict of Interest Statement

The authors declare that the research was conducted in the absence of any commercial or financial relationships that could be construed as a potential conflict of interest.
